# Prevalence of Adverse Events Reported Following the First Dose of COVID-19 Vaccines in Bahia State, Brazil, from 2021 to 2022

**DOI:** 10.3390/vaccines13020161

**Published:** 2025-02-07

**Authors:** Ramon da Costa Saavedra, Enny S. Paixao, Maria Yury Travassos Ichihara, Maria da Conceição Nascimento Costa, Rita Carvalho-Sauer, Caroline Tianeze de Castro, Maria Gloria Teixeira

**Affiliations:** 1 Bahia State Health Department, Salvador 41745-900, BA, Brazil; ritacarvalhosauer@gmail.com; 2 London School of Hygiene and Tropical Medicine, London WC1E 7HT, UK; enny.cruz@lshtm.ac.uk; 3 Center for Data Integration and Knowledge for Health—CIDACS, Salvador 41745-715, BA, Brazil; maria.yury@fiocruz.br; 4 Institute of Collective Health, Federal University of Bahia, Salvador 40110-040, BA, Brazil; mariacncosta@hotmail.com (M.d.C.N.C.); carolinetianeze@gmail.com (C.T.d.C.); t.gloria@hotmail.com (M.G.T.)

**Keywords:** pandemics, COVID19, vaccines, drug-related side effects and adverse reaction, mass vaccination, immunization

## Abstract

**Background**: Despite adverse events following immunization (AEFI) being well described in vaccine trials, there is a need to produce more real-world data on events supposedly attributed to vaccination against COVID-19. This study aims to estimate the prevalence of AEFI in the first dose of COVID-19 vaccines in the state of Brazil and to verify whether such events differ among the types of vaccines offered in this country. **Methods**: A population-based study using linked administrative data on vaccine registry and adverse events following immunization in 2021 and 2022. The study included 10,169,378 individuals aged 18 or over who lived in Bahia and received the first dose of COVID-19 vaccines. We calculated AEFI prevalence and verified differences among vaccines by logistic regression to estimate crude and adjusted by sex and age group prevalence ratio (PR). **Results**: The prevalence of AEFI was 74.3 per 100,000 doses applied, with a higher rate of nonserious events, mainly following the ChAdOx1-S. More than two-thirds of these adverse effects occurred in women, and almost half were between 30 and 49 years old. The individuals who received ChAdOx1-S had a 125% higher prevalence than those who received CoronaVac. Those who received BNT162b2 and Ad26.COV2.S had a 71% and 58%, respectively, lower prevalence of AEFI than those who received CoronaVac. **Conclusions**: The use of vaccines against COVID-19 has proven to be positive and effective in combating SARS-CoV-2, significantly reducing morbidity and mortality from the disease. We cannot deny the presence of adverse events in the context of vaccination. However, the vaccines have proven to be safe and reliable. The results of this study offer relevant data that can contribute to the qualification of AEFI pharmacovigilance in Brazil and worldwide.

## 1. Background

The COVID-19 pandemic stimulated the unprecedented development of new vaccines to contain the spread of SARS-CoV-2. Several types of vaccines, with different technological platforms, some of which are already known and others that are new to date, have been implemented and released for use at a surprising speed and quickly applied globally to millions of people [1,2].

Currently, COVID-19 vaccines are based on four main technologies: inactivated SARS-CoV-2 virus vaccine (CoronaVac); recombinant vaccines using an adenovirus viral vector expressing the SARS-CoV-2 spike protein (ChAdOx1-S and Ad26.COV2.S); and messenger RNA vaccines encoding the SARS-CoV-2 protein (BNT162b2) and protein subunit vaccines, which use SARS-CoV-2 spike protein nanoparticles (Novavax, Gaithersburg, MD, USA). In Brazil, the National Health Surveillance Agency (ANVISA/Agência Nacional de Vigilância em Saúde) authorized the use of the first three technologies mentioned [3,4].

Although adverse events following immunization (AEFIs) are well described in vaccine trials, post-approval surveillance is essential for monitoring the safety of new vaccines. Hence, there is a need for active pharmacovigilance, which allows the estimation of patterns of occurrence and the assessment of the risks and benefits of the use of immunizers on a large scale [5]. Several studies have shown that the occurrence of adverse events varies by vaccine technology. Even so, there is a need to produce more real-world data on AEFIs reportedly attributed to vaccination against COVID-19, as well as comparative analysis of adverse events from vaccines that use different technologies [6,7]. Elucidating the relationship between vaccine types and adverse events following immunization is important for ensuring vaccine safety and, therefore, increasing confidence and acceptance by the general public.

This study aimed to estimate the prevalence of adverse events following the first dose of the COVID-19 vaccine in Bahia, Brazil, and to verify whether such events differ among the types of vaccines offered in this country.

## 2. Methods

### 2.1. Study Design, Location and Period

We conducted a population-based study using linked administrative data on vaccine registries and adverse events following immunization (AEFIs) from January 2021 to December 2022 in Bahia.

Bahia State is located in the Northeast Region of Brazil, being the fifth state in territorial extension (564,760.429 km^2^), occupying 6.6% of the country’s geographic area and 36.34% of the region. It comprises 417 municipalities and is also the fifth largest state in terms of population, with an estimated population of 14,141,626 and a demographic density of 25.04 inhab/km^2^. The distribution of the population in the territory is heterogeneous, with highly populated areas such as the East Macroregion, which accounts for 31.6% of the population, and others where only 5.5% of people live (Extreme South Macroregion). Bahia has a human development index (HDI) of 0.691 and a monthly household income per capita of US$20,296 [8].

Vaccination against COVID-19 in Bahia began in January 2021, with the arrival of the first doses of the CoronaVac and ChAdOx1-S vaccines, initially targeted at priority groups such as healthcare professionals, institutionalized elderly people, and people over 90 years of age, with a gradual expansion of younger age groups until reaching elderly people over 60 years of age. With the arrival of the BNT162b2 vaccine in May 2021 and Ad26.COV2.S in June of the same year, it was possible to expand vaccination to other groups, such as people with comorbidities, pregnant women, and postpartum women. In the second half of 2021, vaccination was opened to the general population between 18 and 59 years of age [9].

### 2.2. Study Population

This study included 10,169,378 individuals aged 18 years or older who lived in Bahia and received the first dose of any COVID-19 vaccine approved for use in Brazil between 2021 and 2022, equivalent to 91.7% coverage of the total number of individuals in this age group [9]. In this population, we identified 7854 reported cases of AEFI in the same period.

### 2.3. Data Sources

We used data from two health information systems: the National Immunization Program Information System (SIPNI) and the Adverse Event Following Immunization Notification System (e-SUS Notifica).

The SIPNI consolidates individualized data on vaccine doses administered nationwide and presents relevant information about the vaccinated person, such as sex, age, race/color, and city of residence. This information system also provides data about the applied vaccine, such as type, dose number and date and place of application [10].

The e-SUS Notifica is a platform created to notify and monitor suspected cases of SARS-CoV-2. It includes data related to adverse events supposedly attributed to vaccination, allowing the follow-up of cases through important demographic and clinical information about the individuals, such as the presence of comorbidities and the characteristics of the side effects presented during the AEFI [11].

### 2.4. Data Linkage Process

The Brazilian Ministry of Health’s Information Technology Department provided data with common unique identifiers that we used to link individual-level records from the two databases. Initially, we searched the SIPNI database, which contains 34,675,000 records of the doses applied, and the e-SUS Notifica database, which contains data on 17,226 adverse events.

**Figure 1:**
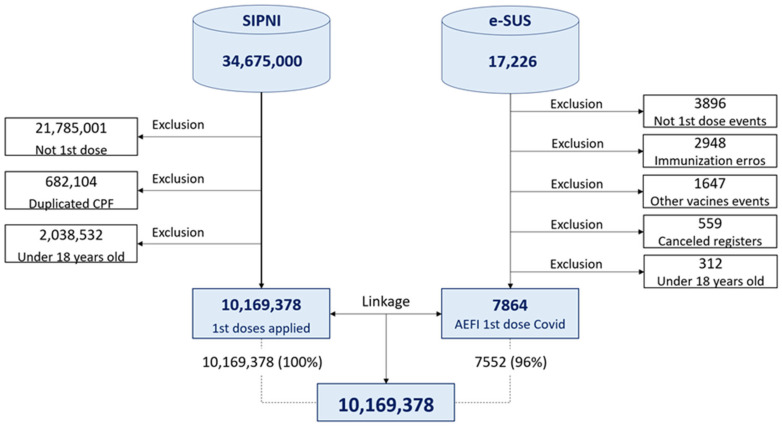
Flowchart of the database composition for the study on the occurrence of adverse events following immunization with the first dose of the COVID-19 vaccine in Bahia, Brazil, 2021–2022. Sources: SIPNI (National Immunization Program Information System in Brazil) and e-SUS Notifica (Information System for Reporting AEFIs in Brazil), 2021–2022.

Records of administered doses that did not refer to the first dose and that occurred in individuals under 18 years of age were excluded. We also excluded records of first doses associated with the same Individual Registration Number (Cadastro de Pessoa Física/CPF), as they were considered duplicates. After this procedure, we arrived at 10,169,378 individuals aged 18 or older, residing in Bahia, who received the first dose of one of the COVID-19 vaccines approved for use in Brazil between 2021 and 2022. Regarding adverse events, records not related to COVID-19 vaccines, not corresponding to the first dose, referring to individuals under 18 years of age and duplicate or canceled notifications were excluded. We also excluded immunization errors, as they were due to inadequate immunization practices and not to problems inherent to the vaccine itself. Thus, 7854 AEFI were identified (Figure 1).

We linked the data from these information systems through the following common identifiers: CPF, National Health Service (Cartão Nacional de Saúde/CNS), in Portuguese) user number, full name, date of birth, mother’s name and municipality of residence. We obtained agreement between 7552 e-SUS Notifica records in the SIPNI, corresponding to 96.0% of the adverse events recorded (Figure 1).

### 2.5. Exposure and Outcomes

The occurrence of adverse events, as defined by the Pan American Health Organization (PAHO) [12], following the first dose of the COVID-19 vaccine was the outcome, and the type of vaccine applied was the main exposure variable.

AEFI is any unwanted or unintended medical occurrence reported after vaccination and does not necessarily have a causal relationship with the use of a vaccine. A severe AEFI is any clinically relevant event that requires hospitalization, that causes risk of death or requires immediate clinical intervention to prevent death, that causes permanent disability, that results in a congenital anomaly, or that causes death [12].

For adequate surveillance, it is essential to adhere to the defined timeframes for characterizing AEFI according to the nature of the event. Immediate reactions, such as anaphylaxis, typically occur within the first 24 h post-vaccination. Early reactions, such as fever, are more common up to the seventh day, and rare events, like thrombosis, may appear within 30 days of vaccination. Moreover, specific neurological conditions require ongoing monitoring and investigation beyond these periods, especially in severe cases [12].

In this study, we included only adverse event records related to the first dose of the COVID-19 vaccine, as it triggers the primary immune response. Focusing on the first dose reduces data variability, especially in populations with different vaccines and dosing schedules. This approach ensures more consistent analysis, yielding reliable results. Additionally, the first dose was widely administered in a short period at the start of the vaccination campaign, providing a robust basis for analysis.

### 2.6. Variables

We selected the variables sex (female, male) and age group (18–29, 30–39, 40–49, 50–59, 60–69, 70–79, 80 or more), which were considered potential confounding variables. We also selected variables related to the place where the event was reported, categorized as macroregion of residence, namely, Central East, Central North, East, Extreme South, North, Northeast, South, Southwest, and West. The vaccine-related variables included the type of vaccine (CoronaVac, ChAdOx1-S, BNT162b2, Ad26.COV2.S) and the date of administration. To characterize the adverse events, we included the variables type (local or systemic), severity (severe or not severe), outcome (recovery or death), the interval in days between vaccine administration and the onset of adverse events (less than 1, 1 day, from 2 to 4 days, 5 or more), and signs/symptoms (locals: pain, swelling, abscess, redness, induration; systemic events: headache, chills, diarrhea, fever, myalgia, fatigue, seizure, thrombotic events, myocarditis/pericarditis).

### 2.7. Statistical Analyses

We estimated the absolute and relative frequency of sociodemographic variables of the studied population and verified the existence of statistically significant differences between strata of variables using the chi-square test.

We calculated the overall AEFI prevalence as the total number of adverse events related to the first dose of the COVID-19 vaccine per 100,000 first doses administered. The proportion of severe AEFIs was estimated by dividing the number of severe events by the total number of adverse events overall and by vaccine type, multiplied by 100.

To assess differences in outcomes associated with different types of COVID-19 vaccines, we applied logistic regression to estimate crude and adjusted (by sex and age group) Odds ratios (Ors) and 95% confidence intervals (95% CIs), taking the CoronaVac vaccine as a reference. The dependent variable in the model was AEFI (Yes/No), and the independent variables were vaccine received (CoronaVac, ChAdOx1-S, BNT162b2, Ad26.COV2.S), age group (18–29, 30–39, 40–49, 50–59, 60–79, 80 and more), and sex (Female, Male).

Bivariate logistic regression was performed to obtain crude ORs. In the multiple logistic regression model, all variables were included simultaneously to obtain adjusted ORs. Logistic regression is widely used in epidemiological studies to model the association between exposure factors and binary outcomes, such as the occurrence or non-occurrence of adverse events following vaccination. This method is based on the logistic function, which describes the probability of an event occurring as a function of independent variables, allowing for adjustment for multiple confounding factors [13]. Its general formula is expressed as:

$$\mathrm{logit}(P)=ln(\frac{P}{1-P})=\beta_{0}+\beta_{1}X_{1}+\beta_{2}X_{2}+\cdots +\beta_{k}X_{k}$$

where *P* represents the probability of the event of interest, *β*_0_ is the intercept (baseline log odds), *β*_1_, *β*_2_, …, *β_k_* are the coefficients of the independent variables *X*_1_ *X*_2_, …, *X_k_*, and each *β*_i_ reflects the effect of a one-unit increase in the variable *X*_i_ on the log odds of the outcome, holding the other variables constant. The uncertainty associated with the estimates of *β*_i_ is expressed by the standard errors (*SE*(*β*_i_)), which are used to construct confidence intervals. For each variable *X*ᵢ, the estimate of *β*_i_ is accompanied by a confidence interval constructed as *β*_i_ ± *Z* · *SE*(*β*_i_), where *Z* is the critical value of the standard normal distribution for the chosen significance level.

All analyses were considered to have a significance level of 5% and were conducted using software version 4.1.3 (R, R Foundation for Statistical Computing, Vienna, Austria).

## 3. Results

After linkage, we obtained a study population consisting of 10,169,378 individuals aged 18 years or older who received the first dose of the COVID-19 vaccine in the state of Bahia from 2021 to 2022. We also identified 7552 adverse events after vaccination specifically related to this population, indicating a prevalence of 74.3 per 100,000 thousand first doses applied.

We observed that 52.9% of the vaccinated population was female, but among those who reported an adverse event, the proportion of females was considerably greater, at 72.2% (*p* < 0.001). Regarding age, 45.4% of people who received the first dose of the vaccine were between 18 and 39 years old, while among individuals who had AEFI, there was a predominance of adults aged between 30 and 49 years (46.9%) (Table 1).

The distribution of the 10,169,378 first doses applied in relation to vaccine types was as follows: 3,839,160 doses (37.8%) were attributed to the ChAdOx1-S vaccine, 3,097,290 doses (30.5%) to the CoronaVac vaccine, 2,948,142 doses (29.0%) to the BNT162b2 vaccine, and 269,276 doses (2.6%) corresponded to the Ad26.COV2.S vaccine. There was no identification of the type of vaccine for 15,510 (0.1%) records of doses administered.

**Figure 2:**
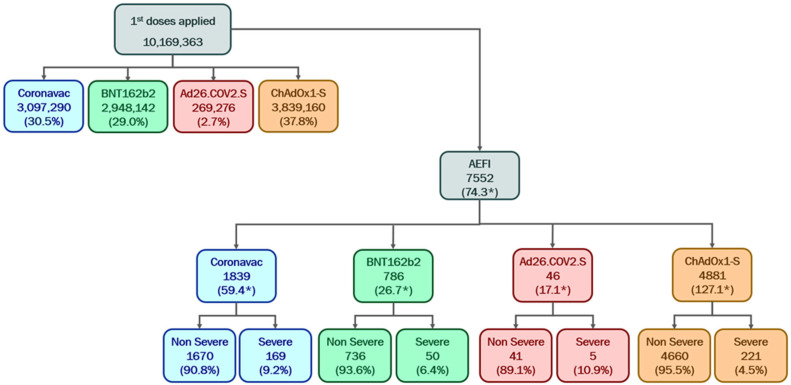
Number and percentage of first applied doses of the COVID-19 vaccine according to the type of vaccine and severity of the adverse events followed by immunization (AEFI). State of Bahia-Brazil, 2021–2022. Sources: SIPNI/e-SUS Notifica EAPV. * per 100,000 doses applied. Note: 15,510 (0.15%) records without identifying the type of vaccine administered.

**Figure 3:**
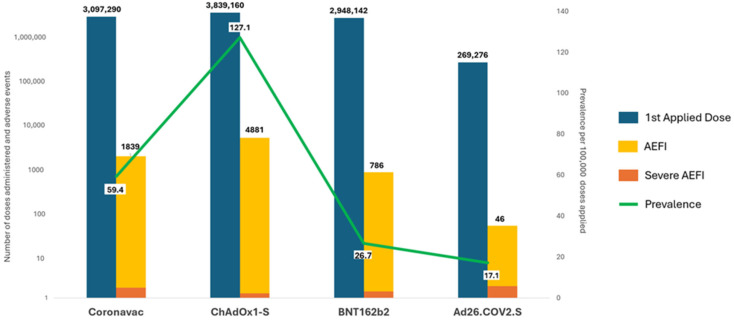
Number of first doses of the COVID-19 vaccine administered, adverse events following immunization (AEFI) and prevalence per 100,000 doses applied, according to type of vaccine. State of Bahia-Brazil, 2021–2022. Sources: SIPNI/e-SUS Notifica EAPV.

Of the 7552 recorded adverse events, 4881 were associated with the ChAdOx1-S vaccine, corresponding to 127.1 events per 100,000 first doses administered. For the CoronaVac vaccine, 1839 adverse events were documented, translating to 59.4 events per 100,000 doses. Similarly, the BNT162b2 vaccine accounted for 786 adverse events, equating to 26.7 events per 100,000 doses. Lastly, the Ad26.COV2.S vaccine reported 46 adverse events, with a prevalence of 17.1 per 100,000 doses administered (Figure 2 and Figure 3).

When characterizing adverse events according to the type of occurrence, a predominance of systemic events was observed in analyses, both overall and stratified by type of vaccine. In the 7552 AEFI reports, considering that an individual may present more than one clinical manifestation, 15,081 events were detected, of which 14,008 (92.9%) were systemic and 1073 (7.1%) were local. Regarding local reactions, the most common were pain (53.5%) and edema (22.1%). Among systemic events, headache (22.6%) and fever (18.2%) were more frequent. Regarding the time between the date of vaccine administration and the onset of the adverse event, 29.5% either started on the same day of vaccine administration, and 27.8% manifested after 5 days or more from the administration date (Table 2).

During the study period, we identified 55 thrombotic events as serious adverse effects reported after the first dose of COVID-19 vaccines. Of these, 50 (90.9%) were related to the ChAdOx1-S vaccine. We also identified nine cases of myocarditis and/or pericarditis, predominantly in men (88.9%) and with a mean age of 30.5 years. Seven (77.8%) of these notifications were related to the BNT162b2 vaccine, while the other two were linked to the CoronaVac and ChAdOx1-S vaccines (Table 2).

The prevalence of AEFI per 100,000 doses of COVID-19 vaccine administered was greater (18.51) among individuals aged 30–39 years and lower (3.04) among adults aged 80 years or older. In the multiple logistic regression model, with AEFI as the outcome and type of vaccine as the main exposure, adjusted for age group and sex, we found that compared to individuals aged 19–29 years, individuals aged 30–39 years had an 8% greater, while individuals aged 50–59 and 60–79 years had 40% and 19% lower chance of AEFI, respectively. Males had a 56% lower chance of having an AEFI than females. Regarding vaccine type, individuals who received ChAdOx1-S had a 125% greater chance of AEFI than those who received CoronaVac. A total of 71% and 58% of those who received BNT162b2 and Ad26.COV2.S, respectively, had a lower incidence of AEFI than those who received the first dose of CoronaVac (Table 3).

Sensitivity analysis were consistent with those found in the main data analysis (Table 3), except for age groups 60 years and more and receiving the Ad26.COV2.S vaccine.

## 4. Discussion

The prevalence of AEFI in individuals aged 18 years and over after the first dose of COVID-19 vaccines was 74.3 per 100,000 thousand doses applied in Bahia from 2021 to 2022. More than two-thirds of these adverse effects occurred in women, and almost half were between 30 and 49 years old. Our study also revealed a greater incidence of nonserious adverse events, mainly following the ChAdOx1-S vaccine. This vaccine also concentrated the majority of reported cases of convulsion and thrombotic events.

Vaccines are produced through an extremely careful quality control process, which guarantees the safety and effectiveness of their large-scale use. Nevertheless, side effects can occur. These effects may be related to the type of vaccine administered, the way the vaccine is produced, the clinical or biological conditions of the person who received the dose and the way the dose was administered. However, most of these events tend to be mild.

The findings of this study are similar to those of an online cohort study with people aged 18 and over in the USA and a retrospective study in Nigeria indicating that younger age and female sex had greater chances of events following the COVID-19 vaccine [14,15]. A cohort study with primary data from seven European countries and a large-scale community survey in the United Kingdom also showed significantly greater reactogenicity in individuals who received a dose of ChAdOx1-S [16,17].

The results of our study in Bahia are very close to the AEFI monitoring carried out by the Ministry of Health in Brazil in this same period, which indicated that the ChAdOx1-S vaccine presented higher incidences of adverse events, mainly in the 18 to 39 age groups and females [18]. ChAdOx1-S is based on an adenovirus from another animal that has been weakened and genetically modified. This technology can promote greater stimulation of the immune system, in addition to a potential adverse response. However, in most cases, these reactions are mild and reversible [3].

The finding that the BNT162b2 vaccine presents a lower prevalence of AEFI among the vaccines offered in Bahia may be related to the fact that these vaccines contain ionizable lipids in their composition to stabilize mRNA, which may also have some adjuvant properties [19]. On the other hand, this was the vaccine administered to more than two-thirds of reported cases of pericarditis, one of the most serious types of AEFI recorded in the study population.

We found a predominance of headache, fever, and myalgia among nonserious adverse events, which was different from the results of a meta-analysis that reported fatigue as the most common mild reaction. On the other hand, fever and myalgia were cited as common adverse events in other scientific articles [20]. The pharmacovigilance of these types of reactions is a complex task due to the enormous volume of cases, and many people choose not to report them because they believe the symptoms will resolve on their own. This leads to underreporting of AEFIs [21].

Regarding serious events, we noticed higher proportions of occurrence in people vaccinated with CoronaVac and Ad26.COV2.S. The interpretation of these findings requires a careful and critical look, as the incorporation of different types of vaccines in Brazil occurred gradually, starting with CoronaVac, which was the first vaccine available in the country and was initially recommended for the most vulnerable groups (elderly people and people with comorbidities), who are also at greater risk of serious illnesses. The Ad26.COV2.S vaccine is occasionally offered in this country, representing less than 3% of the total doses administered [3,4,19].

The thrombotic events identified in this study are mostly related to the ChAdOx1-S vaccine, and these findings are similar to those observed in the context of vaccination against COVID-19 around the world [22]. A study encompassing 99 million vaccinated individuals and 240 million doses administered across 10 countries identified a higher incidence of thrombotic events following the first dose of the ChAdOx1-S [23].

Despite these concerns, the Brazilian Ministry of Health advised that individuals who experienced thrombosis or thrombocytopenia after receiving a first dose of viral vector vaccines, such as ChAdOx1-S, should not receive a second dose of the same platform. Later updates recommended that individuals aged 18 to 39 years receive mRNA-based vaccines instead. However, the overall vaccination guidance remained unchanged, as the low risk of these rare adverse events supports the conclusion that the benefits of vaccination far outweigh the associated risks [24,25].

Effectiveness studies indicate that vaccines administered in Brazil reduce the risk of infection, hospitalization and death from COVID-19 [26]. Furthermore, for each death due to a serious adverse event with a causal relationship compatible with vaccination, approximately 50 thousand other deaths were prevented by vaccination [27].

The investigation of severe events temporarily associated with vaccination against COVID-19 in Bahia was carried out by a specialized committee composed of leading professionals in immunization, infectious disease prevention and epidemiological surveillance [28]. The existence of this department provides greater reliability to the data released and, consequently, to the data presented in our study. Furthermore, this study relied on robust databases, which made it possible to observe AEFI records at the population level.

This study has limitations that should be considered when interpreting the results. First, most adverse events were self-reported, which may introduce significant information bias. The subjective nature of the reports may lead to inaccurate descriptions due to recall bias or symptom interpretation. This bias may also contribute to the underreporting of adverse events, especially of lesser severity, which are often not considered relevant by individuals to be reported. In addition, carrying out a mass vaccination campaign influences the volume of adverse events reported since a greater number of people are exposed to vaccines. This scenario may increase the perception of risk in the population, exacerbating attention to symptoms that do not necessarily have a causal relationship with vaccination^.^ Another important limitation was the lack of data on comorbidities and other sociodemographic characteristics, such as race/color, which are potential confounding variables. Finally, differences observed in the prevalence of adverse events between populations may be related to regional or demographic characteristics that were not fully explored in this study [29,30].

It is important to emphasize that the highest volume of first doses of the COVID-19 vaccine administered in Brazil occurred in 2021 and 2022 when vaccination coverage for this dose reached nearly 100%. We extracted data up to June 2024; however, no reports of adverse events following the first dose in adults were found after 2022. Furthermore, the mRNA-1273 vaccine was not available in Brazil during the study period and, therefore, was not included in the investigation.

## 5. Conclusions

The occurrence of side effects after immunization cannot be completely excluded; however, they are rare events that pose significantly fewer threats than complications of the disease itself [30,31]. Nevertheless, we recognize the importance of combining research to investigate the long-term safety of vaccines against COVID-19, especially because they are new vaccines.

The use of vaccines against COVID-19 has proven to be effective in combating severe acute respiratory syndrome coronavirus 2 (SARS-CoV-2), significantly reducing morbidity and mortality from the disease. We cannot deny the presence of adverse events in the context of vaccination; however, these vaccines have proven to be safe and reliable. The results of this study offer relevant data that can contribute to the understanding of AEFI pharmacovigilance in Brazil and worldwide.

## Figures and Tables

**Table 1:** Number and percentage of first applied doses of the COVID-19 vaccine and adverse events following immunization (AEFI) according to sociodemographic characteristics of the vaccinated population. State of Bahia-Brazil, 2021–2022.

Sociodemographic Characteristics		Applied Doses		AEFI (Yes)		AEFI (No)		*p*
		N	%	N	%	N	%	
Total		10,169,378	100.0	7552	100.0	10,161,826	100.0	
Sex								<0.001
	Female	5,383,752	52.9	5456	72.2	5,378,296	52.9	
	Male	4,785,603	47.1	2096	27.8	4,783,507	47.1	
	Missing	23	0.0	...	...	23	0.0	
Age group (years)								<0.001
	18–29	2,455,809	24.1	1471	19.5	2,454,338	24.2	
	30–39	2,169,250	21.3	1882	24.9	2,167,368	21.3	
	40–49	2,001,065	19.7	1663	22.0	1,999,402	19.6	
	50–59	1,551,612	15.3	1035	13.7	1,550,577	15.3	
	60–79	1,674,562	16.5	1192	15.8	1,673,370	16.5	
	≥80	317,065	3.1	309	4.1	316,756	3.1	
	Missing	23	0.0	…	…	23	0.0	
Macroregion								<0.001
	East	3,268,100	32.1	3160	41.8	3,264,940	32.1	
	East Center	1,421,230	14.0	767	10.2	1,420,463	14.0	
	Extreme South	489,263	4.8	202	2.7	498,061	4.8	
	North	656,269	6.5	218	2.9	656,051	6.5	
	North Center	511,285	5.0	485	6.4	510,800	5.0	
	North East	532,217	5.2	538	7.1	531,679	5.2	
	South	1,007,764	9.9	1025	13.6	1,006,739	9.9	
	South West	1,166,355	11.5	734	9.7	1,165,621	11.5	
	West	586,548	5.8	133	1.8	586,415	5.8	
	Others States	449,740	4.4	251	3.3	449,489	4.4	
	Missing	80,607	0.8	39	0.5	80,568	0.8	

Source: SIPNI (National Immunization Program Information System in Brazil) and e-SUS Notifica (Information System for Reporting AEFI in Brazil), 2021–2022.

**Table 2:** Number and percentage of adverse events followed by immunization (AEFI) of first-dose COVID-19 vaccines, according to the type of vaccine and selected characteristics of these events. State of Bahia-Brazil, 2021–2022.

Vaccines		CoronaVac		ChAdOx1-S		BNT162b2		Ad26.COV2.S	
Characteristics of Adverse Events		N	%	N	%	N	%	N	%
Total		1839	24.4	4881	64.6	786	10.4	46	0.6
Type									
	Local	220	20.5	693	64.6	152	14.2	8	0.7
	Systemic	3596	25.7	8630	61.6	1738	12.4	44	0.3
Severity									
	Nonsevere	1670	23.5	4660	65.6	736	10.4	41	0.5
	Severe	169	38.0	221	49.7	50	11.2	5	1.1
Outcome									
	Cure	275	15.3	1353	75.5	150	8.4	14	0.8
	Death	36	42.4	42	49.4	6	7.1	1	1.1
	Follow-up	24	10.9	156	70.9	38	17.3	2	0.9
	Missing	1504	27.6	3330	61.0	592	10.9	29	0.5
Time to onset (in days)									
	0	373	16.7	1637	73.4	215	9.6	4	0.3
	1	269	22.7	799	67.3	111	9.4	8	0.6
	2–4	78	23.1	234	69.2	22	6.5	4	1.2
	5 or more	522	24.9	1375	65.5	191	9.1	12	0.5
	Missing	597	35.2	836	49.2	247	14.5	18	1.1
Main local adverse events									
	Pain	108	18.8	420	73.2	42	7.3	4	0.3
	Edema	47	19.0	160	23.7	28	40.7	2	0.7
	Abscess	4	16.0	18	72.0	3	12.0	-	-
	Redness	8	22.9	13	37.1	13	37.1	1	2.9
	Induration	19	40.4	25	56.8	2	4.5	1	2.3
Main systemic adverse events									
	Headache	757	24.0	2059	65.2	331	10.4	13	0.4
	Chills	87	9.9	682	77.3	110	12.5	3	0.3
	Diarrhea	227	36.9	334	54.3	54	8.8	-	-
	Fever	338	13.3	1960	77.1	228	9.0	17	0.6
	Myalgia	312	18.2	1215	70.9	180	10.5	6	0.4
	Fatigue	180	22.0	530	64.6	110	13.4	-	-
	Convulsion	7	25.0	18	64.3	3	10.7	-	-
	Thrombotic events	1	1.8	50	90.9	4	7.3	-	-
	Pericarditis	1	11.1	1	11.1	7	77.8	-	-

Source: e-SUS Notifica.

**Table 3:** Prevalence (per 100,000 applied doses) and odds ratio (OR), crude and adjusted, of adverse events following the first dose of different COVID-19 vaccine types. State of Bahia-Brazil, 2021–2022.

Variables	Prevalence per 100,000 Applied Doses	Odds Ratios	
		Crude OR [95% CI]	Adjusted OR ^1^ [95% CI]
Vaccines			
CoronaVac	59.4	1.00	1.00
ChAdOx1-S	127.1	**2.14 [2.03; 2.26]**	**2.25 [2.13; 2.38]**
BNT162b2	26.7	**0.29 [0.22; 0.39]**	**0.29 [0.22; 0.40]**
Ad26.COV2.S	17.1	**0.45 [0.41; 0.49]**	**0.42 [0.38; 0.46]**
Age group (years)			
18–29	14.47	1.00	1.00
30–39	18.51	**1.45 [1.35; 1.55]**	**1.08 [1.01; 1.16]**
40–49	16.65	**1.38 [1.29; 1.49]**	0.93 [0.89; 1.01]
50–59	10.18	**1.11 [1.03; 1.21]**	**0.60 [0.55; 0.65]**
60–79	11.72	**1.19 [1.10; 1.28]**	**0.71 [0.66; 0.77]**
80 and more	3.04	**1.63 [1.44; 1.84]**	1.01 [0.89; 1.14]
Sex			
Female	53.65	1.00	1.00
Male	20.61	**0.43 [0.41; 0.45]**	**0.44 [0.42; 0.46]**

^1^ adjusted by sex and age. Note: Bold text highlights values whose confidence interval does not include 1.

## Data Availability

The datasets generated during and/or analyzed during the current study are available from the corresponding author upon reasonable request.

## References

[1] Chung J.Y., Thone M.N., Kwon Y.J. (2021). COVID-19 vaccines: The status and perspectives in delivery points of view. Adv. Drug Deliv. Rev..

[2] Hasan T., Beardsley J., Marais B.J., Nguyen T.A., Fox G.J. (2021). The Implementation of Mass-Vaccination against SARS-CoV-2: A Systematic Review of Existing Strategies and Guidelines. Vaccines.

[3] Ndwandwe D., Wiysonge C.S. (2021). COVID-19 vaccines. Curr. Opin. Immunol..

[4] Brazil. Ministry of Health (MS) (2021). National Operationalization Plan for COVID-19 Vaccination.

[5] Rudolph A., Mitchell J., Barrett J., Sköld H., Taavola H., Erlanson N., Melgarejo-González C., Yue Q.Y. (2022). Global safety monitoring of COVID-19 vaccines: How pharmacovigilance rose to the challenge. Ther. Adv. Drug Saf..

[6] Romero-Ibarguengoitia M.E., González-Cantú A., Pozzi C., Levi R., Mollura M., Sarti R., Sanz-Sánchez M.Á., Rivera-Salinas D., Hernández-Ruíz Y.G., Armendariz-Vázquez A.G. (2022). Analysis of immunization time, amplitude, and adverse events of seven different vaccines against SARS-CoV-2 across four different countries. Front. Immunol..

[7] Kant A., Jansen J., van Balveren L., van Hunsel F. (2022). Description of Frequencies of Reported Adverse Events Following Immunization Among Four Different COVID-19 Vaccine Brands. Drug Saf..

[8] IBGE (2022). Brazilian Institute of Geography and Statistics. Demographic Census 2022 [Internet].

[9] Brazil Ministry of Health National Health Data Network. COVID-19 Vaccination Panel. https://infoms.saude.gov.br/extensions/SEIDIGI_DEMAS_Vacina_C19/SEIDIGI_DEMAS_Vacina_C19.html.

[10] Silva B.S., de Azevedo Guimarães E.A., de Oliveira V.C., Cavalcante R.B., Pinheiro M.M., Gontijo T.L., Rodrigues S.B., Ferreira A.P., de Oliveira Quites H.F., Pinto I.C. (2020). National Immunization Program Information System: Implementation context assessment. BMC Health Serv. Res..

[11] Brazil Ministry of Health e-SUS Notifica: Epidemiological Surveillance Information System [Internet]. https://www.gov.br/saude/pt-br/composicao/svsa/sistemas-de-informacao/e-sus-notifica.

[12] PAHO (2022). Manual for Surveillance of Events Supposedly Attributable to Vaccination or Immunization in the Region of the Americas.

[13] Kleinbaum D.G., Kupper L.L., Chambless L.E. (1982). Logistic regression analysis of epidemiologic data: Theory and practice. Commun. Stat.—Theory Methods.

[14] Beatty A.L., Peyser N.D., Butcher X.E., Cocohoba J.M., Lin F., Olgin J.E., Pletcher M.J., Marcus G.M. (2021). Analysis of COVID-19 Vaccine Type and Adverse Effects Following Vaccination. JAMA Netw. Open.

[15] Odeigah L.O., Mutalub Y.B., Agede O.A., Obalowu I.A., Aiyetoro S., Jimoh G.A.A. (2022). Adverse events following COVID-19 vaccination in Kwara State, North-central Nigeria. PLoS Glob. Public Health.

[16] Raethke M., van Hunsel F., Thurin N.H., Dureau-Pournin C., Mentzer D., Kovačić B., Mirošević Skvrce N., De Clercq E., Sabbe M., Trifirò G. (2023). Cohort Event Monitoring of Adverse Reactions to COVID-19 Vaccines in Seven European Countries: Pooled Results on First Dose. Drug Saf..

[17] Menni C., Klaser K., May A., Polidori L., Capdevila J., Louca P., Sudre C.H., Nguyen L.H., Drew D.A., Merino J. (2021). Vaccine side-effects and SARS-CoV-2 infection after vaccination in users of the COVID Symptom Study app in the UK: A prospective observational study. Lancet.

[18] Brazil Ministry of Health, Health Surveillance Secretariat (2023). Monitoring the safety of COVID-19 vaccines in Brazil until epidemiological week no. 11 of 2023. Epidemiol. Bull..

[19] Akaishi T., Onodera T., Takahashi T., Harigae H., Ishii T. (2023). Acute Adverse Events at a Mass Vaccination Site after the Third and Fourth COVID-19 Vaccinations in Japan. Tohoku J. Exp. Med..

[20] Haas J.W., Bender F.L., Ballou S., Kelley J.M., Wilhelm M., Miller F.G., Rief W., Kaptchuk T.J. (2022). Frequency of Adverse Events in the Placebo Arms of COVID-19 Vaccine Trials: A Systematic Review and Meta-analysis. JAMA Netw. Open.

[21] Heininger U., Holm K., Caplanusi I., Bailey S.R., CIOMS Working Group on Vaccine Safety (2017). Guide to active vaccine safety surveillance: Report of CIOMS working group on vaccine safety—Executive summary. Vaccine.

[22] Simpson C.R., Shi T., Vasileiou E., Katikireddi S.V., Kerr S., Moore E., McCowan C., Agrawal U., Shah S.A., Ritchie L.D. (2021). First-dose ChAdOx1 and BNT162b2 COVID-19 vaccines and thrombocytopenic, thromboembolic and hemorrhagic events in Scotland. Nat. Med..

[23] Faksova K., Walsh D., Jiang Y., Griffin J., Phillips A., Gentile A., Kwong J.C., Macartney K., Naus M., Grange Z. (2024). COVID-19 vaccines and adverse events of special interest: A multinational Global Vaccine Data Network (GVDN) cohort study of 99 million vaccinated individuals. Vaccine.

[24] Goddard K., Lewis N., Fireman B., Weintraub E., Shimabukuro T., Zerbo O., Boyce T.G., Oster M.E., Hanson K.E., Donahue J.G. (2022). Risk of myocarditis and pericarditis following BNT162b2 and mRNA-1273 COVID-19 vaccination. Vaccine.

[25] Brazil Ministry of Health (2023). Anvisa Clarifies About the Risk of Myocarditis and Pericarditis After Vaccination.

[26] Freitas C.M.D., Barcellos C., Villela D.A.M., Matta G.C., Reis L.C., Portela M.C., Xavier D.R., Guimarães R., Saldanha R.F., Mefano I.V. (2022). Fiocruz COVID-19 Observatory Bulletin: Special Bulletin: Two-Year Balance of the COVID-19 Pandemic: January 2020 to January 2022. https://portal.fiocruz.br/documento/boletim-covid-balanco-de-2-anos-da-pandemia.

[27] Watson O.J., Barnsley G., Toor J., Hogan A.B., Winskill P., Ghani A.C. (2022). Global impact of the first year of COVID-19 vaccination: A mathematical modelling study. Lancet Infect. Dis..

[28] Bahia. Secretaria da Saúde. Diretoria de Vigilância Epidemiológica. Portaria Interna nº 01, de 11 de Fevereiro de 2021. Institui e Nomeia Membros para Compor a Câmara Técnica para Análise dos Eventos Adversos Pós-Vacinação Graves, Temporalmente Associados à Vacina Contra a COVID-19, no âmbito da Diretoria de Vigilância Epidemiológica da Secretaria da Saúde do Estado da Bahia. Diário Oficial do Estado da Bahia. Salvador, BA; 18 fev. 2021. https://dool.egba.ba.gov.br/.

[29] Dhamanti I., Suwantika A.A., Adlia A., Yamani L.N. (2023). Adverse Reactions of COVID-19 Vaccines: A Scoping Review of Observational Studies. Int. J. Gen. Med..

[30] Chen R.T., Rastogi S.C., Mullen J.R., Hayes S.W., Cochi S.L., Donlon J.A., Wassilak S.G. (1994). The Vaccine Adverse Event Reporting System (VAERS). Vaccine.

[31] Correa I., Pereira M.A., Luz K.C.S.I., Teixeira M.M., Santos F.G.T. (2022). Adverse reactions after COVID-19 vaccination: An integrative review. Res. Soc. Dev..

